# Differences in the Tongue Features of Primary Dysmenorrhea Patients and Controls over a Normal Menstrual Cycle

**DOI:** 10.1155/2017/6435702

**Published:** 2017-05-31

**Authors:** Jihye Kim, Haebeom Lee, Hyunho Kim, Jong Yeol Kim, Keun Ho Kim

**Affiliations:** ^1^KM Fundamental Research Division, Korea Institute of Oriental Medicine, 1672 Yuseongdae-ro, Yuseong-gu, Daejeon, Republic of Korea; ^2^Department of Biofunctional Medicine and Diagnosis, College of Korean Medicine, Sangji University, 83 Sangjidae-gil, Wonju-si, Gangwon-do, Republic of Korea; ^3^Department of Human Informatics of Korean Medicine, Interdisciplinary Programs, Kyung Hee University, 26 Kyungheedae-ro, Dongdaemun-gu, Seoul, Republic of Korea; ^4^Department of Biofunctional Medicine & Diagnostics, College of Korean Medicine, Kyung Hee University, 26-6 Kyungheedae-ro, Dongdaemun-gu, Seoul, Republic of Korea

## Abstract

**Background:**

The aims of this study were to investigate the relationships between tongue features and the existence of menstrual pain and to provide basic information regarding the changes in tongue features during a menstrual cycle.

**Methods:**

This study was conducted at the Kyung Hee University Medical Center. Forty-eight eligible participants aged 20 to 29 years were enrolled and assigned to two groups according to their visual analogue scale (VAS) scores. Group A included 24 females suffering from primary dysmenorrhea (PD) caused by qi stagnation and blood stasis syndrome with VAS ≥ 4. In contrast, Group B included 24 females with few premenstrual symptoms and VAS < 4. All participants completed four visits (menses-follicular-luteal-menses phases), and the tongue images were taken by using a computerized tongue image analysis system (CTIS).

**Results:**

The results revealed that the tongue coating color value and the tongue coating thickness in the PD group during the menstrual phase were significantly lower than those of the control group (*P* = 0.031 and *P* = 0.029, resp.).

**Conclusions:**

These results suggest that the tongue features obtained from the CTIS may serve as a supplementary means for the differentiation of syndromes and the evaluation of therapeutic effect and prognosis in PD.

**Trial Registration:**

This trial was registered with Clinical Research Information Service, registration number KCT0001604, registered on 27 August 2015.

## 1. Introduction

Primary dysmenorrhea (PD) is a common gynecological disorder. Menstrual pain typically begins during or just prior to menses [1, 2]. The prevalence of PD is highest in adolescent girls and women of reproductive age, with estimates ranging from 20 to 90% [3]. In Korea, 78.3% of all adolescent girls have dysmenorrhea [4].

The most common treatments for PD are hormone therapy, nonsteroidal anti-inflammatory drugs (NSAIDs), prostaglandin synthesis inhibitors, and oral contraceptive pills. However, these treatments have temporary effects and may cause unwanted menopausal symptoms, such as sweating, hot flashes, vaginal dryness, dyspareunia, breast reduction, and decreased sexual desire [5–8]. For the aforementioned reasons, PD patients turn to alternative therapies in many countries [9].

Traditional Korean medicine (TKM) is known to provide more satisfactory results for patients because it employs different targeted therapies for each patient. Korean medical doctors (KMDs) focus on the PD subtype, namely, the* zheng* or syndrome, for example, cold-dampness stagnation or qi stagnation and blood stasis syndrome [7, 10]. The syndrome guides the choice of treatment with acupuncture, moxibustion, or herbal medicine. Therefore, correct syndrome identification is the most important clinical process [2, 4, 8, 10].

Each syndrome is identified by KMDs based on the clinical information obtained through the four main diagnostic procedures in TKM: inspection (望), the listening/smelling examination (聞), inquiry (問), and palpation (切) [11].

Tongue diagnosis is one of the four diagnostic methods, which is a part of inspection. Changes in tongue features provide significant information for TKM diagnosis in clinical practice. According to TKM theory, the changes in the tongue body (color, shape, and movement) and tongue coating (color, thickness, moisture, and distribution) are crucial components of the tongue diagnostic method. The tongue features are used to diagnose imbalances in the essential components, such as qi, blood, yin, and yang, and to determine whether the patient has a heat or cold syndrome. Therefore, tongue features reveal the state of organ functions, the balance of qi and blood, the progression of health conditions, and illness severity [12, 13].

Physiological parameters change throughout a menstrual cycle and depend on the presence or absence of menstrual pain. Therefore, studies of adolescent girls and women of reproductive age should consider the effects of these factors [14]. Previous research studies have compared the tongue colors of healthy subjects in the luteal and follicular phases [14]. However, no published reports exist regarding the differences in tongue features between healthy subjects and PD patients across the menstrual cycle.

The aims of this study were as follows: (1) to investigate the relationships between tongue features based on the existence or nonexistence of menstrual pain and (2) to provide basic information about changes in tongue features based on the effects of the physiological menstrual cycle.

## 2. Methods

### 2.1. Hypotheses

The hypotheses of this study were as follows: (1) tongue features will differ significantly between PD patients and controls and (2) tongue features will differ across the menstrual, follicular, and luteal phases of the menstrual cycle.

### 2.2. Study Design

This clinical study was conducted at a single center as a prospective and observational study. The entire process was performed at the Kyung Hee University Korean Medicine Hospital (KHUKMH) in Seoul, Republic of Korea, from June 2015 to January 2016.

### 2.3. Participants

The participants were recruited through posters displayed around Kyung Hee University and KHUKMH. All participants visited KHUKMH and were asked to sign informed consent forms after receiving a full explanation of the study.

This study enrolled 48 women aged 20 to 30 years who were subsequently stratified into two groups according to the visual analogue scale (VAS). The VAS for the evaluation of menstrual pain was used to distinguish between PD (VAS score ≥ 4) and non-PD (VAS score < 4). The VAS is commonly used to measure the intensity of menstrual pain. The VAS consists of a 10 cm horizontal line with endpoints of “no pain” on the far left and the “worst possible pain” on the far right. All participants were asked to indicate pain intensity scored from a minimum of zero to a maximum of ten.

This study focused on PD with qi stagnation and blood stasis syndrome because the most common factor causing dysmenorrhea is blood stagnation in the uterus [15, 16]. Furthermore, PD may exhibit different patterns with respect to syndrome differential and diagnosis, and different patterns may be correlated with different tongue characteristic changes. By performing this study on PD patients with specific patterns, we minimized the differences due to varying patterns and sought to identify characteristic tongue features of PD patients with qi and blood stagnation. Two KMDs diagnosed the participants to determine whether PD was caused by qi and blood stagnation.

The participants meeting the following criteria were included: (1) females between 20 and 30 years of age; (2) menstrual cycle lasting 28 ± 3 days for the prior 3 months (excluding menstrual irregularities or missed menses); (3) a VAS score from 4 to 10 (PD patients) or a VAS score from 0 to 4 (controls); (4) the ability to communicate with the clinical study researchers and complete the questionnaires; (5) the ability to voluntarily agree to participate in this clinical study; and (6) the ability to provide written informed consent.

The participants with one or more of the following criteria were excluded: (1) secondary dysmenorrhea caused by uterine myoma, endometriosis, or infection of the genitals confirmed by an ultrasound exam; (2) medical operations or procedures; (3) a history of major neuropsychiatric disorder or antidepressant, anti-serotonin agent, barbiturate, or psychotropic drug use within the prior 3 months; (4) a history of major medical diseases (e.g., hypertension, diabetes, hyperlipidemia, gastritis, enteritis, gastroesophageal reflux disease, and Crohn's disease); (5) extreme dieting within the prior week; (6) pregnancy (as determined by a urine pregnancy test), planned pregnancy, or lactation; (7) congenital vascular anomalies or a history of wrist fracture; (8) a history of another clinical study in the previous month; and (9) exclusion at the investigator's discretion [17].

### 2.4. Sample Size

The calculation of the required sample size was guided by a previous study [17]. With a level of statistical significance of 0.05 (2-sided test) and a study power of 80%, the estimated sample size was calculated as 19 participants per group (patient and healthy subject groups) by using the R statistical environment, a free software program for statistical computing and graphics. Based on a dropout rate of 20%, 23.75 subjects should be recruited for each group. Consequently, the total sample size was 48 with a 1 : 1 ratio [17].

### 2.5. Experimental Procedures

**Figure 1:**
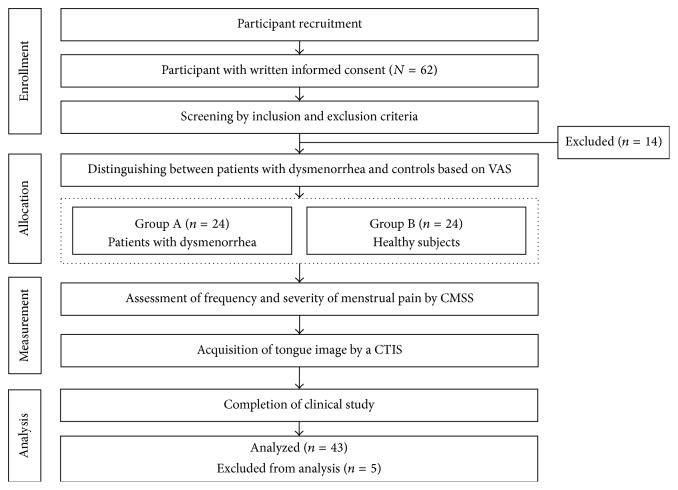
Flow diagram of the clinical study.

Figure 1 presents the experimental procedures. An investigator surveyed the demographic characteristics. After completing a screening test, the allocation process was performed, and the 48 enrolled participants were divided into two groups.

Normally, the menstrual cycle length is 28 days. The entire duration of a menstrual cycle can be divided into four main phases: (1) the follicular phase (days 6 to 13); (2) the ovulation phase (day 14); (3) the luteal phase (days 15 to 28); and (4) the menstrual phase (days 1 to 5).

After completing the allocation process, a research investigator calculated the assessment period of each subject considering menstrual cycle length for the prior 3 months. Upon completion of the run-in period, the assessments were conducted during the follicular (9th day of the menstrual cycle) and luteal phases (22nd day of the menstrual cycle), as well as at the beginning of the next menstrual phase [18].

A practitioner who is also a licensed physician evaluated the outcome variables as an assessor and recorded vital signs and any adverse events during the assessment period. The patients sent the relevant information to the practitioner immediately.

### 2.6. Outcome Assessments

#### 2.6.1. Tongue Colors and Tongue Coating Thickness Assessment

**Figure 2:**
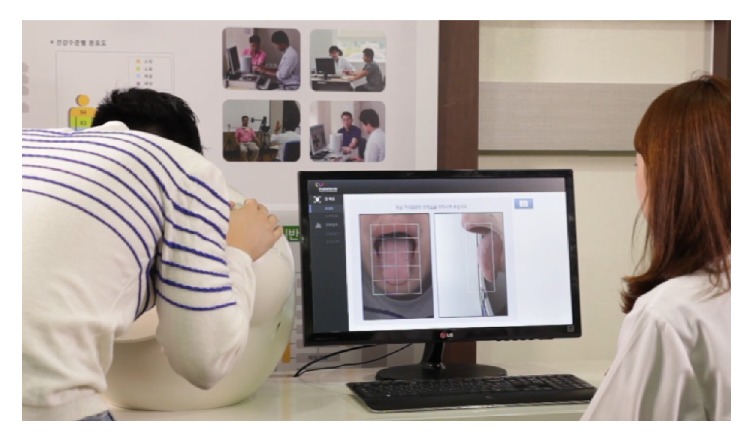
Acquisition of a tongue image.

The outcomes were the changes in the tongue features captured by the computerized tongue image analysis system (CTIS) across the follicular, luteal, and menstrual phases. The investigational CTIS was developed by the Korean Institute of Oriental Medicine (TAS-4000, Korea Institute of Oriental Medicine, Daejeon, Republic of Korea). The CTIS components and image analysis have been described previously in detail [19].  Figure 2 shows the acquisition of a tongue image.

Multiple factors, such as lighting sources (e.g., illumination, color temperature, and color rendering index) and test room conditions (e.g., temperature, humidity), can influence tongue features. Therefore, these variables were assessed and controlled throughout the study too. Also, the participants were requested to comply with the following criteria: (1) to avoid using any oral rinse or breath freshener for 1 week before the experiment day; (2) to avoid drinking caffeine, alcohol, or milk and smoking 24 h before the experiment; (3) to abstain from eating and drinking for at least 8 h before the experiment, although they were allowed to drink water up to 3 h before the experiments. Therefore, we minimize the influence of multiple factors. Additionally, the CTIS used in this study was developed to minimize the inflow of external light using an ergonomic facing part [19].

**Figure 3:**
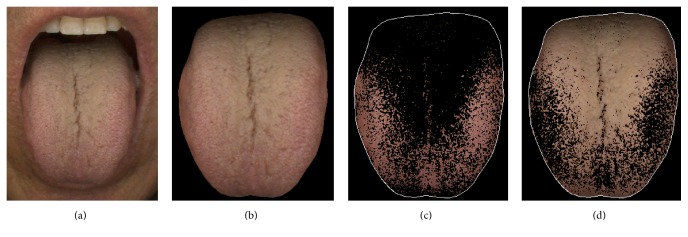
Illustrations show (a) an original tongue image, (b) segmented tongue region, (c) extracted tongue substance, and (d) extracted tongue coating from the tongue region to be acquired with the CTIS. A trained operator acquired the tongue image according to SOPs.

The outcomes were as follows: tongue body color (TBC), tongue coating color (TCC), and TCT. To extract features from the images for statistical analysis, image processing of the 144 images (48 subjects × 3 phases) was performed. From the frontal image, the tongue region was segmented using various techniques (Figure 3) [20, 21].

Pixel values in the red-green-blue (RGB) color space were converted to the Commission Internationale de l'Éclairage (CIE) Lab space, which is a color-opponent space with dimensions *L*^*∗*^ for lightness and *a*^*∗*^ and *b*^*∗*^ for the color-opponent dimensions. The CIE *L*^*∗*^ from the CIE Lab color space is widely used for tongue diagnosis because the CIE *L*^*∗*^ value is appropriate for representing the amount of tongue coating or tongue coating thickness. The CIE *a*^*∗*^ from the CIE Lab color space is appropriate for representing red intensity. The CIE *a*^*∗*^ is lower in the pale red tongue (*淡紅舌*) than in the red tongue (*紅舌*), purple tongue (*紫舌*), or bluish purple tongue (*絳舌*). High-luminance regions, which were caused by light reflection from saliva, were excluded from the analysis.

The CTIS measured the TCT, which is widely used in clinical tongue diagnosis as an objective and useful parameter [22, 23]. The cut-off points that separated no coating (*薄胎*) and thick coating (*厚苔*) from thin coating (normal) were 29.06% and 63.51%, respectively [23].

#### 2.6.2. Cox Menstrual Symptom Scale (CMSS)

The CMSS has been widely used for the integrative evaluation of patient symptoms. The scale consists of 17 items or symptoms. The severity evaluation gives each symptom one of five scores: a score of 0 indicates that the symptom is not noticeable; a score of one indicates that the symptom is slightly bothersome; a score of two indicates the symptom as moderately bothersome; a score of three illustrates that the symptom is severely bothersome; and a score of four indicates that the symptom is very severely bothersome. In the duration evaluation, each symptom was given one of the following five scores: a score of 0 indicates that the symptom did not occur; a score of one indicates that the symptom lasted less than three hours; a score of two indicates that the symptom lasted between three and seven hours; a score of three illustrates that the symptom lasted an entire day; and a score of four indicates that the symptom lasted several days [24].

### 2.7. Statistical Analysis

SPSS version 23.0 was used to analyze the data. Descriptive statistical analyses were performed to explore the data. The level of significance was set to *P* < 0.05 for all the analyses. Statistics experts analyzed the relationship between the results using Pearson's chi-squared test and the independent* t*-test. At the screening stage, fourteen participants who had been diagnosed with PD were excluded based on the inclusion and exclusion criteria. Five subjects were excluded from the analysis due to protocol violation. The data from the remaining 43 participants were used for analysis in this study.

## 3. Results

### 3.1. Comparison of Baseline Characteristics between the Patient and Control Groups

The general characteristics of the patients and controls are presented in Table 1.

There were no significant differences between the two groups with respect to baseline characteristics, including age, height, weight, body mass index, systolic blood pressure, diastolic blood pressure, pulse rate, body temperature, and menstruation characteristics (menses duration and menarche age). However, the VAS revealed a significant difference in the severity of menstrual pain between the PD patients and controls. The CMSS indicated significant differences in the duration and severity of menstrual pain between the PD patients and controls.

### 3.2. Comparison of the Tongue Color Values between the Patient and Control Groups

We compared the TBC parameters of the PD patients with those of the controls (Table 2). There were no significant differences in the CIE *L*^*∗*^, *a*^*∗*^, and *b*^*∗*^ values between the PD patients and the controls throughout the menstrual cycle.

Table 3 presents the results for TCC differences between the patients and controls over the menstrual cycle. The CIE *L*^*∗*^ value in the menstrual phase was significantly lower in the patient group than in the control group (*P* = 0.031). The CIE *b*^*∗*^ value in the luteal phase was significantly higher in the patient group than in the control group (*P* = 0.035).

### 3.3. Comparison of the Tongue Coating Thicknesses between the Patient and Control Groups

No significant differences were observed between the TCT values in the two groups in the follicular and luteal phases. However, Table 4 indicates that the TCT significantly differed between the patient and control groups in the menstrual phase. The total TCT and the TCT at the central area during the menstrual phase were significantly lower in the PD patient group than in the control group (*P* = 0.029 and 0.031, resp.). Although there was no significant difference in the TCTs at the root and tip areas between the two groups, we found that the TCTs of the PD patient group trended lower than those of the control group (*P* = 0.056 and 0.073, resp.). Overall, the PD patients exhibited less TCT than the controls.

### 3.4. Associations between Menstrual Pain and the Tongue Features

Table 5 shows the results associations between menstrual pain and the tongue features. No significant associations were observed between the TBCs and duration of menstrual pain (both VAS and CMSS). However, the severity of menstrual pain on the VAS was significantly associated with *L*^*∗*^ (*P* = 0.006) and *a*^*∗*^ (*P* = 0.019) for tongue body values.

Similarly to the tongue body, no significant associations were observed between the TCCs and the duration of menstrual pain (both VAS and CMSS). However, the severity of menstrual pain on the VAS was significantly associated with *L*^*∗*^ (*P* = 0.007) and *b*^*∗*^ (*P* = 0.039) values of tongue coating.

Similar results were obtained as described above in TBC and TCC. No significant associations were observed between the TCT and duration of menstrual pain (both VAS and CMSS). However, the severity of menstrual pain on the VAS was significantly associated with total TCT (*P* = 0.003), TCT at the root area (*P* = 0.009), TCT at the central area (*P* = 0.003), and TCT at the tip area (*P* = 0.006).

## 4. Discussion

PD is a common gynecological disorder among adolescent girls and women of reproductive age [25]. The most common choices for the treatment of PD are hormone therapy, NSAIDs, prostaglandin synthesis inhibitors, and oral contraceptive pills. However, these treatments have temporary effects and may cause unwanted side effects [5–8].

TKM is believed to provide more satisfactory results for patients because it employs different targeted therapies for each patient. Hence, many patients with PD turn to alternative therapies, such as acupuncture, moxibustion, or herbal medicine [9].

Tongue diagnosis is an important diagnostic method for examining patient conditions in clinical practice. The tongue is closely related to organs such as the heart, spleen, kidney, liver, and lungs through meridians. Therefore, it is thought that tongue features change in the presence of several diseases, including anemia, appendicitis, cancer, diabetes, immune hepatic injury, and gastrointestinal disease [12, 13, 26]. For example, a reddish-purple tongue and thin tongue coating usually indicate blood stasis syndrome or blood stagnation [14, 15].

Hsieh et al. found that the greatest differences in color values between the follicular phase and the luteal phase were observed in healthy subjects with normal cycles [14]. Zhang et al. found that the color of the tongue varied from darker red to pink during the menstrual cycle [27].

The aims of this study were to investigate the relationship between tongue features and the presence or absence of menstrual pain and to provide basic information about the changes in tongue features according to the effects of the menstrual cycle.

To distinguish patients with PD from healthy subjects, the VAS was used as an inclusion criterion during the screening process for this study. A significant difference was observed in the severity of menstrual pain (VAS) between the PD patient and control groups. The CMSS revealed significant differences in the duration and severity of menstrual pain between the PD patient and control groups.

In this study, tongue images of all participants were obtained during the follicular, luteal, and menstrual phases with a CTIS. We compared the tongue features of patients with those of controls.

There were no obvious differences in the CIE Lab values of the tongue body between the two groups throughout the menstrual cycle. Although there was no significant difference in the CIE Lab values between the two groups, we found that the CIE *L*^*∗*^ value tended to be lower in the PD patient group than in the control group and that the CIE *a*^*∗*^ was higher in the PD patient group than in the control group. These findings signify that the tongue body was more reddish in the PD patient group than in the control group. The CIE *L*^*∗*^ of the tongue coating differed significantly between the two groups during the menstrual phase. The CIE *L*^*∗*^ value in the menstrual phase was significantly lower in the PD patient group than in the control group (*P* = 0.031), which indicates that the TCT was thinner in the PD patient group than in the control group.

The total TCT and the TCT at the central area in the menstrual phase of the PD patient group were significantly lower than those of the control group (*P* = 0.029 and *P* = 0.031, resp.). The TCTs at the central and tip areas in the menstrual phase were thinner in the PD group than in the control group, although this difference was not significant (*P* = 0.056 and *P* = 0.073, resp.).

In this study, a significant difference was observed in the TCTs of the two groups. The PD patients had significantly thinner coatings in the menstrual phase. In contrast, no significant differences in the TBC were observed between the two groups. However, we observed a trend for PD patients to exhibit more reddish tongues during the menstrual phase.

No significant associations were observed between the tongue features and duration of menstrual pain on the VAS and the CMSS. No significant associations were observed between the tongue features and the severity of menstrual pain on the CMSS. However, the severity of menstrual pain on the VAS was significantly associated with *L*^*∗*^ and *a*^*∗*^ values of TBC and *L*^*∗*^ and *b*^*∗*^ values of TCC and TCT in all areas. As the severity score of menstrual pain of VAS increased, the TBC became reddish and darker and TCT decreased.

There are four possible reasons why significant differences were observed in the TCTs between the two groups.

First, this study focused on PD with qi stagnation and blood stasis syndrome. Patients with typical blood stagnation symptoms normally exhibit a purplish tongue (which indicates stagnant qi and blood) and thin fur (a tongue coating which is faintly visible on a tongue surface) [14, 15, 28]. Interestingly, the results of this study are consistent with TKM theory.

Second, many studies have suggested that severe menstrual pain is associated with some degree of autonomic imbalance [29–31]. Because the autonomic nervous system dominates the sensory nerves and the movement of the entire gastrointestinal tract, disorders of the autonomic nervous system cause symptoms such as indigestion, appetite changes, vomiting, and diarrhea. Such symptoms might increase or decrease the TCT because TCT is associated with the state of five viscera, particularly stomach function [15, 24, 31]. In addition, some articles suggest that the tongue coating at the central part of the tongue may be associated with the conditions of the spleen and stomach [32–34]. Consistent with this idea, unlike the TCT at the tip and root areas of the tongue, the TCT at the central area was statistically significant.

Third, the microbial flora on the tongue coating represents one of the major microbiomes in the human body and is at the forefront of the human alimentary system [35]. Therefore, TCT may have changed due to symptoms such as indigestion, appetite changes, vomiting, and diarrhea caused by PD.

Fourth, from the perspective of Western medicine, the tongue coating is dependent on the microcirculation, such as fluid circulation, hemodynamic parameters, body temperature, and hormones [36]. According to TKM theory, the most common cause of PD is blood stagnation in the uterus. Blood stagnation, which includes extravasated and congested blood or sluggishly circulating blood, is a very important concept [15]. When the flow of qi and blood is smooth, the body is healthy and free of diseases. However, the interruption of this flow in the uterus causes menstrual pain [15]. A change in the fluid circulation and hemodynamic parameters may have caused the changes in the TCT.

We believe that this study provides good information and a reference for further studies. The tongue features obtained from the CTIS may serve as a supplementary means for the differentiation of syndromes and the evaluation of therapeutic effect and prognosis in PD.

Moreover, the results of this study show that there was a significant difference only in the menstrual phase but not in other phases. These results provide substantial guidance to identify optimal treatment timing for PD in clinics.

This study has two limitations. First, this study was designed as a single-center, prospective, and a cross-sectional study without randomization, blinding, or allocation concealment. Therefore, it might be difficult to confirm the final results of the analysis. Second, this study lacks observations regarding the correlation of tongue features with the other symptoms or syndromes associated with PD. Third, comparisons were performed with a large number of variables including CIE Lab values of tongue body and coating, root-center-tip areas, and four phases using a relative small sample size. This design might lead to type I error, also known as a false positive (incorrectly rejecting the null hypothesis). We will proceed to a large, multicenter trial after deliberating the results of this study.

## 5. Conclusions

Our study is the first to report that tongue features change over a normal menstrual cycle. The results of this study suggest that the TCC obtained from the CTIS may serve as a supplementary means for the differentiation of qi stagnation and blood stasis syndrome and the evaluation of therapeutic effect and prognosis in PD. Moreover, considering that the identification of optimal treatment timing for PD has substantial significance for clinical practice, this study serves as a reference for further studies.

## Figures and Tables

**Table 1:** Comparison of baseline characteristics between the patient and control groups (*N* = 43).

Variable	PD patients (*n* = 19)	Controls (*n* = 24)	*P* value
Age	23.72 ± 2.30	22.91 ± 1.63	0.186
Height	162.56 ± 4.32	160.57 ± 4.41	0.145
Weight	53.10 ± 3.84	52.23 ± 5.50	0.563
Body mass index	20.09 ± 1.17	20.25 ± 1.91	0.751
Systolic blood pressure	105.53 ± 9.29	111.63 ± 10.34	0.051
Diastolic blood pressure	69.26 ± 5.50	73.04 ± 8.20	0.092
Pulse rate	74.32 ± 9.94	79.75 ± 10.70	0.096
Body temperature	36.67 ± 0.25	36.80 ± 0.28	0.120
Menses, duration (days)	29.11 ± 1.76	29.54 ± 1.61	0.403
Age at menarche	13.16 ± 2.71	12.92 ± 1.53	0.714
VAS, duration of menstrual pain	1.11 ± 2.18	0.35 ± 0.94	0.174
VAS, severity of menstrual pain	6.63 ± 1.34	2.13 ± 1.02	0.000^*∗∗*^
CMSS, duration of menstrual pain	26.37 ± 9.77	8.96 ± 6.44	0.000^*∗∗*^
CMSS, severity of menstrual pain	25.74 ± 6.40	8.85 ± 6.02	0.000^*∗∗*^

The data are represented as the means ± standard deviations; VAS: visual analogue scale; CMSS: Cox menstrual symptom scale; ^*∗∗*^*P* < 0.01.

**Table 2:** Comparison of the tongue body colors between the patient and control groups (*N* = 43).

Variable	Phase	PD patients (*n* = 19)	Controls (*n* = 24)	*P* value
*L*	Follicular	52.91 ± 0.48	52.84 ± 0.52	0.925
	Luteal	52.73 ± 0.66	52.83 ± 0.44	0.901
	Menstrual	52.37 ± 0.54	53.46 ± 0.45	0.127
*a*	Follicular	20.82 ± 0.18	20.79 ± 0.16	0.893
	Luteal	21.21 ± 0.24	21.05 ± 0.20	0.610
	Menstrual	21.25 ± 0.26	20.80 ± 0.14	0.112
*b*	Follicular	13.06 ± 0.26	12.37 ± 0.31	0.107
	Luteal	13.51 ± 0.25	12.90 ± 0.32	0.156
	Menstrual	13.21 ± 0.32	12.72 ± 0.34	0.312

The data are represented as the means ± standard deviations.

**Table 3:** Comparison of the tongue coating colors between the patients and controls (*N* = 43).

Variable	Phase	PD patients (*n* = 19)	Controls (*n* = 24)	*P* value
*L*	Follicular	44.45 ± 1.29	45.80 ± 1.12	0.431
	Luteal	42.74 ± 1.70	44.17 ± 0.96	0.468
	Menstrual	41.90 ± 1.29	45.41 ± 0.95	0.031^*∗*^
*a*	Follicular	13.50 ± 0.11	13.40 ± 0.10	0.505
	Luteal	13.55 ± 0.13	13.57 ± 0.11	0.907
	Menstrual	13.60 ± 0.13	13.38 ± 0.11	0.218
*b*	Follicular	11.81 ± 0.33	11.08 ± 0.25	0.077
	Luteal	12.47 ± 0.43	11.37 ± 0.29	0.035^*∗*^
	Menstrual	11.97 ± 0.38	11.06 ± 0.31	0.069

The data are represented as the means ± standard deviations; ^*∗*^*P* < 0.05.

**Table 4:** Comparison of tongue coating thicknesses between the patients and controls (*N* = 43).

Variable	Phase	PD patients (*n* = 19)	Controls (*n* = 24)	*P* value
Total	Follicular	32.54 ± 2.73	35.13 ± 2.23	0.463
	Luteal	27.85 ± 2.98	29.08 ± 2.59	0.757
	Menstrual	25.35 ± 2.70	33.07 ± 2.15	0.029^*∗*^
Root	Follicular	65.02 ± 3.99	66.83 ± 2.91	0.709
	Luteal	57.89 ± 4.48	58.17 ± 3.54	0.960
	Menstrual	54.67 ± 4.62	65.51 ± 3.28	0.056
Center	Follicular	24.69 ± 3.66	29.06 ± 3.17	0.370
	Luteal	19.21 ± 3.55	22.90 ± 3.53	0.472
	Menstrual	16.74 ± 3.03	26.28 ± 2.94	0.031^*∗*^
Tip	Follicular	4.23 ± 0.89	6.13 ± 1.22	0.239
	Luteal	3.73 ± 0.85	4.19 ± 0.98	0.733
	Menstrual	3.01 ± 0.55	4.91 ± 0.80	0.073

The data are represented as the means ± standard deviations; ^*∗*^*P* < 0.05.

**Table 5:** Associations between menstrual pain and the tongue features.

Variable	VAS		CMSS	
	Duration of menstrual pain	Severity of menstrual pain	Duration of menstrual pain	Severity of menstrual pain
Tongue body colors				
*L*	−.130	−.411^*∗∗*^	−.156	−.168
*a*	−.076	.357^*∗*^	.141	.159
*b*	−.063	.187	.184	.179
Tongue coating colors				
*L*	.022	−.402^*∗∗*^	−.260	−.240
*a*	−.071	.241	.020	.087
*b*	.022	.317^*∗*^	.201	.233
Tongue coating thickness				
Total	.053	−.444^*∗∗*^	−.183	−.236
Root	.091	−.394^*∗∗*^	−.116	−.189
Center	.048	−.449^*∗∗*^	−.279	−.293
Tip	−.074	−.414^*∗∗*^	−.252	−.164

^*∗*^ *P* < 0.05, ^*∗∗*^*P* < 0.01.

## Abbreviations

PD: Primary dysmenorrhea
NSAIDs: Nonsteroidal anti-inflammatory drugs
TKM: Traditional Korean medicine
KMD: Korean medical doctors
KHUKMH: Kyung Hee University Korean Medicine Hospital
VAS: Visual analogue scale
CTIS: Computerized tongue image analysis system
TBC: Tongue body color
TCC: Tongue coating color
TCT: Tongue coating thickness
CMSS: Cox menstrual symptom scale
RGB: Red-green-blue
CIE: Commission Internationale de l'Éclairage.
